# Two-dimensional material-based memristive devices for alternative computing

**DOI:** 10.1186/s40580-024-00432-7

**Published:** 2024-06-27

**Authors:** Jey Panisilvam, Ha Young Lee, Sujeong Byun, Daniel Fan, Sejeong Kim

**Affiliations:** Department of Electrical and Electronic Engineering, Faculty of Engineering and Information Technology, University of Melbourne, Melbourne, 3000 Australia

**Keywords:** 2D materials, Memristors, Resistive switching, Neuromorphic computing, Crossbar arrays

## Abstract

Two-dimensional (2D) materials have emerged as promising building blocks for next generation memristive devices, owing to their unique electronic, mechanical, and thermal properties, resulting in effective switching mechanisms for charge transport. Memristors are key components in a wide range of applications including neuromorphic computing, which is becoming increasingly important in artificial intelligence applications. Crossbar arrays are an important component in the development of hardware-based neural networks composed of 2D materials. In this paper, we summarize the current state of research on 2D material-based memristive devices utilizing different switching mechanisms, along with the application of these devices in neuromorphic crossbar arrays. Additionally, we discuss the challenges and future directions for the field.

## Introduction

Computing machines based on the von Neumann architecture and CMOS fabrication technology have been the backbone of the information revolutions for the past 70 years, driving significant societal changes. Despite the widespread use of conventional digital computers in today’s world, future computers for large-scale distributed systems will require the use of increasingly powerful hardware. However, transistors are approaching their fundamental physical limits, posing challenges in continuously increasing transistor density to keep pace with the exponentially growing demand for computing power. Moreover, the physical separation of the central processing unit (CPU) and memory adopted in von Neumann architecture limits computational speed. For these reasons, alternative computing architectures, or non-von Neumann models have been intensively explored, often involving in-memory computing to overcome the problems associated with data traffic between CPU and memory. One essential component is a memristive device, which operates as both a memory and a switch, first proposed by L. Chua in 1971 [1]. Unlike a conventional transistor that operates as a current switch and handles binary information, a memristive device functions as a current switch that remembers the voltage or current that has passed through a device. Memristive devices offer several advantages including non-volatility, low power consumption, and parallelism, allowing for applications such as analog computing [2, 3], in-memory computing [4, 5] and most importantly for the current technological climate, neuromorphic computing [6, 7]. The underlying mechanism varies depending on the type of memristor, but it generally involves the movement of atomic defects or ions within the device structure, often leading towards a dielectric material being placed between two conductive plates. By applying a controlled voltage or current, modulation of the resistance can occur effectively programming the device’s resistance and storing information.

While memristance is a well-studied phenomenon in bulk materials, only recently has there been extensive research into memristive devices using 2D materials. 2D materials provide a significant advantage over bulk materials due to several reasons. Firstly, the ability to construct monolayers to multilayers with high precision makes the device highly configurable, because the bandgap of 2D materials vary with the number of layers [8]. A larger bandgap leads to a smaller switching voltage and a higher switching ratio of the created devices [9, 10], indicating the capability of engineering these devices by controlling the layer thickness. For example, one piece of research has developed methods to control layer thickness directly to improve the performance of resistive switching in 2D materials [11]. Secondly, due to reduced thickness between two electrodes, 2D material-based memristors tend to be more sensitive to external stimuli such as light or strain. For example, one recent research paper has demonstrated manipulating synaptic properties of memristors through the use of varying compressive strains [12]. Other research utilizes the strain sensitivity to manipulate and store resistance values for downstream applications such as gesture recognition [13]. Lastly, 2D material-based memristors consume lower power due to their thinness, resulting in lower operating voltages, which is crucial for addressing heating-related problems and scaling up integration density. There have been several successful demonstrations of low-power operating memristors through the use of nanosheets for mature 2D materials such as WS_2_ [14] and MoS_2_ [15], and these results have also shown to be scalable to large crossbar arrays [16–19] and other 2D materials such as HfSe_2_, SnS, and PdSe_2_ [20–23].

In this review, we summarize recent efforts to demonstrate alternative computing paradigms using 2D materials to create devices that differ from the traditional von Neumann architecture. We first introduce 2D materials that possess resistive switching behaviors and their advantages when used, followed by an overview of resistive switching mechanisms, including conductive filament formation, phase transition and charge trapping, and their contribution to developing neuromorphic computing devices. This is then followed by introducing applications of memristors in neuromorphic computing, and by discussing large-scale integration and scalability via crossbar arrays. We conclude by discussing the potential impact of these technologies on future computing devices.

## 2D materials for memristors

**Fig. 1:**
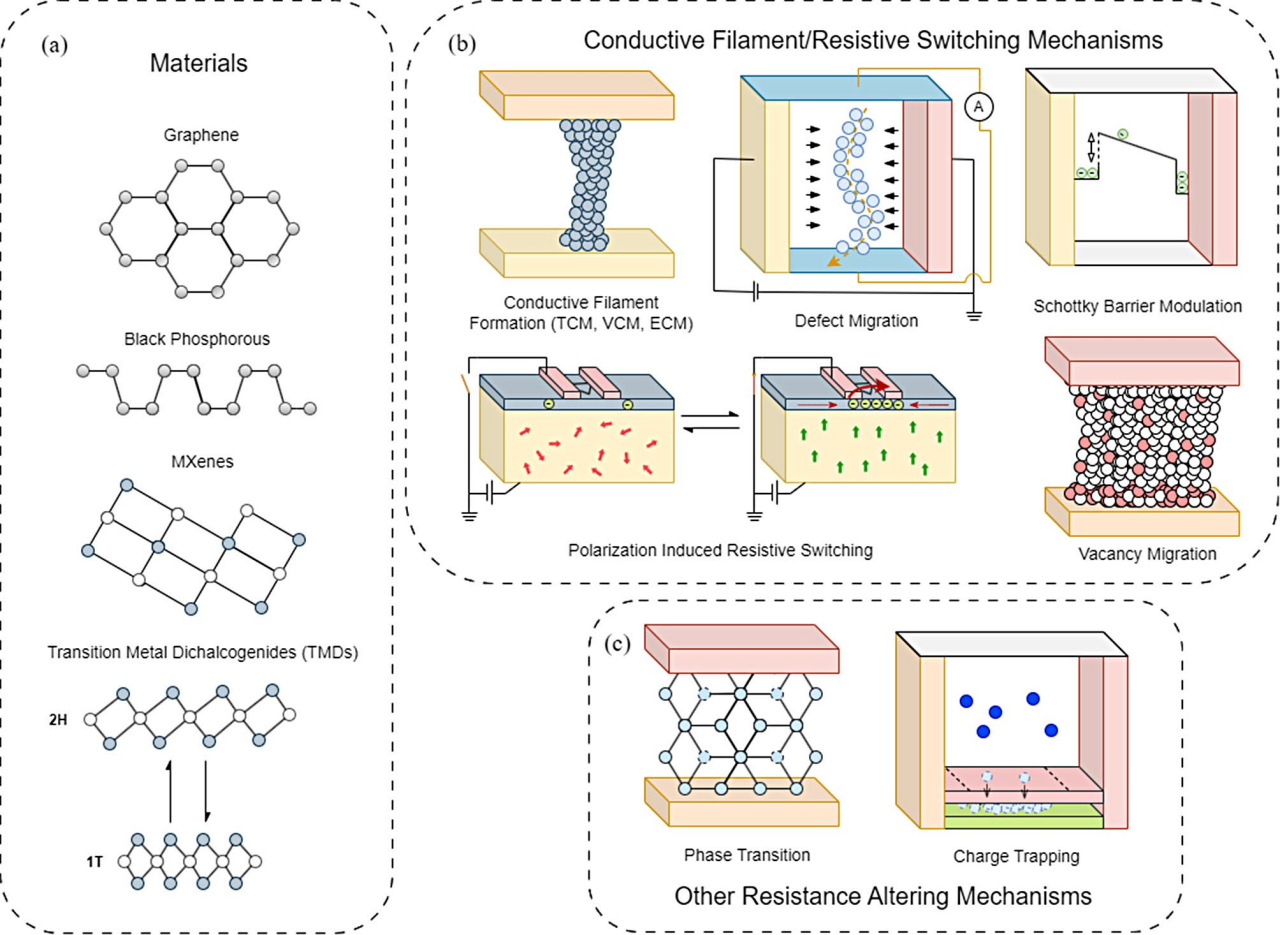
2D material memristive devices. (**a**) Candidate 2D materials. (**b**) Visual representation of major resistance altering mechanisms. (**c**) Resistance altering mechanisms that do not generally form conductive filaments in order to alter resistance values

Although bulk material-based memristors offer the benefits of stable retention of memory states and endurance over many switching cycles, they face challenges in achieving precise control over their decay time, ON/OFF ratios, switching times, scalability, and energy efficiency. Deploying 2D materials can address these issues. Figure 1a shows representative materials with strong optical and electronic effects that are suitable for the development of 2D material-based resistive switching devices: graphene, black phosphorous (BPs), MXenes, and transition metal dichalcogenides (TMDs) [24–35]. Below is a summary of each material.

Graphene is a desirable material for memristive devices mainly due to its low contact resistance and its overall stability during operation [36–38]. Optically, graphene exhibits interactions with light from ultraviolet to the far infrared [39, 40], as well as waves in the terahertz and microwave regions [41]. Additionally, it possesses a linear energy-momentum dispersion relation [42]. Graphene has excellent electrical conductivity [43], and there have been several studies reporting the tunability of graphene’s electrical and optical properties through the use of dopants [44]. Black phosphorous provides a precise level of tunability in its bandgap and carrier mobility by changing the number of layers present in its structure [45, 46]. Optically, it absorbs and emits wavelengths between mid-infrared to the visible spectrum [47]. Despite the instability of black phosphorous in air [48], its anisotropic properties for light absorption due to its atomic structure allow it to be utilized in applications such as optical modulators and photodetectors [41, 49]. Both graphene and black phosphorous have been used extensively in resistive switching devices in the past [29, 43, 50–53]. MXenes refer to the family of transition metal carbides, nitrides, and carbonitrides constructed in 2D forms. MXenes hold an advantage over well-known materials such as graphene because it is significantly easier to attach other materials on their surface due to the stronger interlayer bonds formed from hydrogen bonding, electrostatic interactions, and coordination bonds [54, 55]. Another advantage of MXenes is shown by its high electrical conductivity at 10,000 $S c{m}^{-1}$, while also possessing high optical transmittance [56, 57]. Having both these properties allows for in-circuit manipulation of MXene elements, creating more degrees of freedom in applications. MXenes have also been shown to have nonlinear optical responses in the broadband [58–61]. Due to the aforementioned properties, in literature they have proven to be excellent memristive devices [33, 62]. TMDs are atomically thin semiconductor materials. Monolayer TMDs exhibit a direct bandgap, a characteristic that differs from their bulk counterparts, where the bandgap is indirect. TMD devices with an odd number of layers have exhibited even-order nonlinearities such as second harmonic generation [63–65], which is attributed to the broken inverse symmetry [41]. More recently, third order nonlinearities have also been demonstrated in these devices as well [66].

Another advantage of using 2D materials is the easy construction of van der Waals (vdW) heterostructures, which harnesses the unique benefits of 2D materials while alleviating fabrication limitations. Until recently, creating heterostructures for resistive switching has been limited due to issues such as lattice mismatch, complexities in switching mechanisms unique to heterostructure configurations, and a limited understanding of the interactions between materials within a heterostructure. In recent years, a better understanding of 2D materials has enabled progress in this field due to important results in making heterostructures that present strong nonvolatile switching devices [67–78]. To incorporate 2D materials into practical devices, devices are usually constructed in either a lateral or vertical structure, and individual monolayers are almost always stacked vertically on top of each other.

## Switching mechanisms

Resistive switching involves altering the electrical resistance of a device through applied external stimuli, in contrast to classical components that maintain a fixed resistance value. Materials demonstrating such behavior are typically limited to metal-insulator-metal oxides or transition metal oxides [79, 80] in bulk materials, often displaying hysteresis that leads to memristive behavior within these devices. In recent years, 2D materials have also been shown to exhibit resistive switching [81–83], including optical resistive switching [84–86] and hybrid optoelectronic resistive switching [86, 87], which could provide significant benefits through the manipulation of electrons using light.

Explanations for the underlying mechanisms governing resistive switching have been proposed, but there are some features that are universal to all material types. Resistive switching consists of switching devices between two different resistive states: a High Resistive State (HRS) and a Low Resistive State (LRS). The LRS makes a device act as a conductor, and the process of moving from a HRS to a LRS is referred to as a ‘SET’ operation. The reverse process is referred to as a ‘RESET’ operation. Within this convention, there are also two switching modes referred to as bipolar and unipolar switching [88]. Bipolar devices have a polarity-dependent switching characteristic, while unipolar devices can enter the ‘SET’ or ‘RESET’ states regardless of the polarity of the applied potential. This results in the unipolar ‘SET’ or ‘RESET’ operation being purely dependent on the magnitude of the applied energy. In certain cases, unipolar and bipolar resistive switching can exist in the same device, and hence the presence of either resistive switching mode is not mutually exclusive [89–91]. In addition, the presence of a ‘SET’ or ‘RESET’ process at a certain point in time does not necessarily ensure that the states will persist in the future [92–94]. As a result, there have been several key metrics developed to determine how well a resistive switching device will perform in practice. These include ON/OFF ratio which is a measure of the contrast between a HRS and LRS, where a higher ratio is more desirable as it indicates a clearer distinction between the two states, along with the endurance of switching cycles over time, a retention time of a ‘SET’ or ‘RESET’ state, switching speed, and energy consumption [95, 96].

Various mechanisms to induce resistive switching have been explored so far as summarized in Fig. 1b and c. Those include conductive filament formation [97–104], vacancy migration [105–107], and polarization-induced resistive switching [73, 74]. More recently, there have been resistive switching effects that have been found that are specific to 2D materials, namely Schottky emission [108, 109], direct tunnelling [26, 110], defect migration [27, 111, 112], and certain phase transition effects [113, 114]. Besides the mechanisms that enable resistive switching, the construction of the resistive switching structure also contributes to its overall performance.

Multi-level states are a desired characteristic for memristors, and this has been explored in previous work on resistive switching devices. Charge trapping [115, 116], magneto resistive effects, and phase transition effects [117] are mechanisms that change a device between several levels of resistance states without forming a conductive filament. Of these effects, phase transition has garnered significant attention due to its energy efficiency, cycle endurance, switching speed, thermal stability, and multi-level storage through intermediate states between amorphous and crystalline phases [74, 76]. Both magneto resistive and charge trapping mechanisms are established technologies [118, 119], however their use in 2D material devices is complex, and often involve hybrid devices, incorporating bulk materials and quantum dots [74, 120–125].

In the following subsections, detailed working principles behind memristive switching will be discussed, along with future methods for representation of information, as well as material composition and methods of construction of resistive switching devices.

### Conductive filament formation

There are three accepted mechanisms for resistive switching through conductive filament formation: thermochemical mechanisms (TCM) [126–130], valence change mechanisms (VCM) [88, 131, 132], and electrochemical metallization (ECM) [133, 134]. These mechanisms all modulate resistance through controllable conductive filament formation, but the nature of the change differs. TCM relies of Joule-heating induced modifications, VCM involves the migration of oxygen vacancies or ions, and ECM involves the formation and dissolution of metallic filaments due to ion migration.

ECM devices consist of dielectric layers sandwiched between a top electrode made from an active metal and a bottom electrode made of an inert metal. When a positively biased voltage is applied, metal cations are generated by the oxidization reaction and driven to the bottom electrode [133]. After the cations move through the insulating layer, along the existing electric field, they accumulate and form a conductive filament consisting of reduced metal atoms or clusters [100, 135]. When the anode and cathode have a direct connection, resistance drops resulting in the HRS turning into an LRS. The reverse process can occur by applying an opposite voltage causing an oxidation reaction and rupturing the conductive filaments where they eventually migrate back to their previous unstructured positions. In 2019, a vertical MoS_2_ double-layer memristor using ECM as the main mechanism was reported by Xu and colleagues [136]. The double layered MoS_2_ was sandwiched between a copper (Cu) top electrode and a gold (Au) bottom electrode. The Cu ions migrate through the MoS_2_ double layers to form atomic-scale filaments, which enables the switch between resistive states. In their study, they observed a low SET voltage of 0.25 V which is attributed to the low energy barrier required for Cu ions to diffuse into the MoS_2_ layer, as well as the thin nature of memristive medium, leading to reduced distance between the electrodes. They also demonstrated analog switching behavior, indicating that it can take multiple resistive states between its HRS and LRS. This feature is important for simulating synaptic functions in neuromorphic computing for example.

For VCM devices, both electrodes are made of inert metals, and the dielectric layer is a transition metal oxide. When a voltage is applied across the electrodes, an electric field is generated, and the cations within the metal undergo reversible oxidation and reduction reactions. As a result of these redox reactions, conductive filaments are formed within the insulating layer via the migration of oxygen vacancies, allowing a larger or smaller flow of electrical current between the electrodes [102, 133]. TCM refers to the ability of a resistive switching device to respond to thermal fluctuations, which can cause ion migration through thermochemical reactions. Devices that are dominated by TCM display unipolar switching characteristics [137], where switching behavior is caused by an increase in local temperature gain above which conductive filaments are ruptured or formed. This can be utilized to reset a resistive switching device. Both VCM and TCM have been demonstrated with 2D materials such as graphene oxide (GO) [138], tungsten diselenide (WS_2_) [139], and hexagonal boron nitride (hBN) [140].

### Vacancy migration

**Fig. 2:**
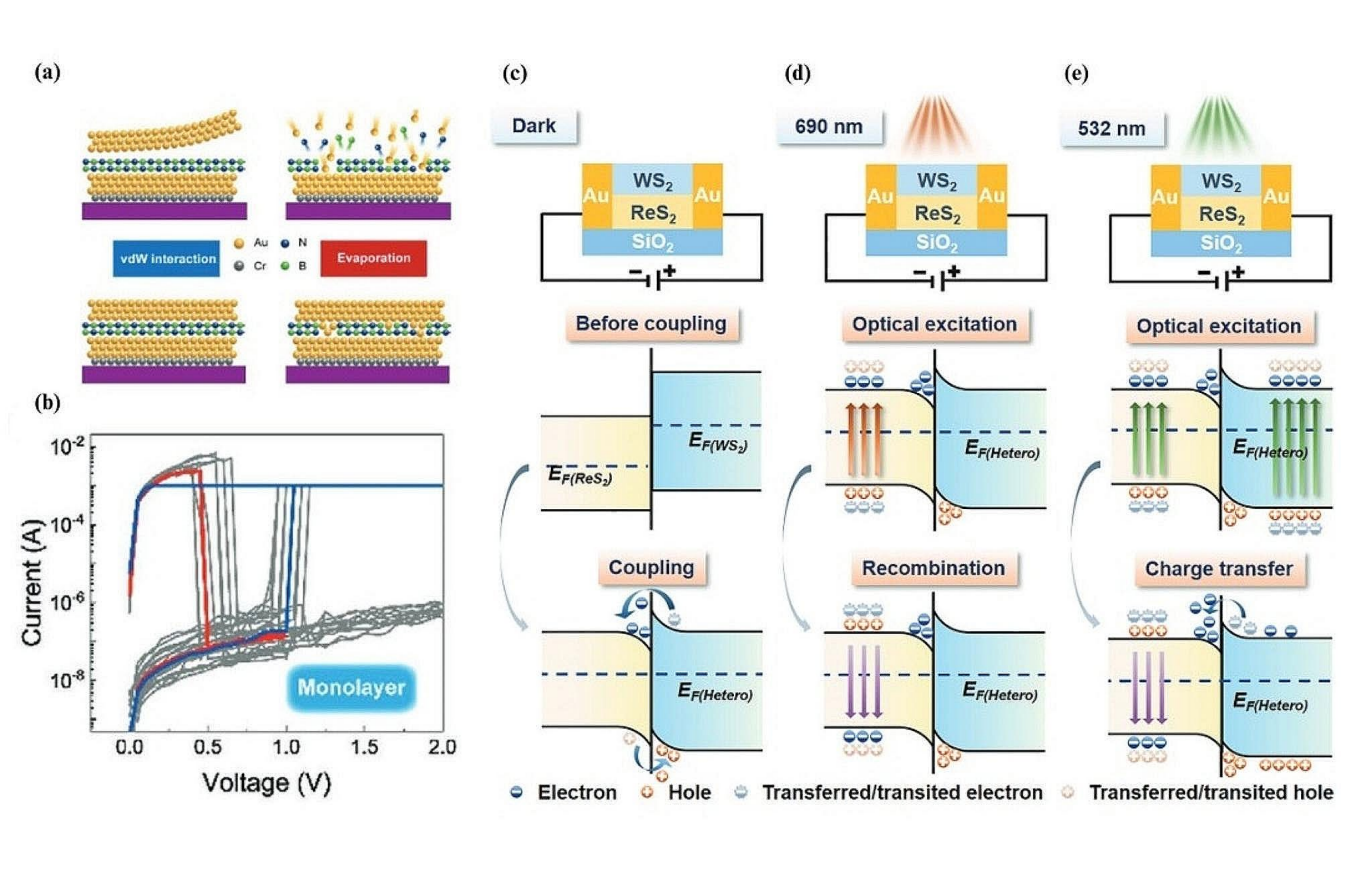
Vacancy migration in 2D material memristive devices. (**a**) Schematic of the top electrode integration on vdW materials using the transfer process (left) and evaporation (right). (**b**) Resistive switching properties of a hBN monolayer with exfoliated manufacturing process. The SET process of the device is shown in blue, and the RESET process of the device is shown in red. The cycles of SET and RESET are shown in gray to illustrate the device variability. (**c**) Schematic diagram and band structure process of ReS_2_/WS_2_ heterostructure under no optical modulation. (**d**) Demonstration of optical excitation under 690 nm light, where recombination occurs after optical excitation. (**e**) Demonstration of optical excitation under 532 nm light which reduces the SET voltage of the device. Figure 2. (**a, b**) Reproduced with permission [109]. Copyright 2022, Wiley-VCH GmbH. Figure 2. (**c-e**) Reproduced with permission [142]. Copyright 2023, The Authors. Advanced Science published by Wiley‐VCH GmbH

Vacancy migration is one of the first memristive mechanisms that was successfully demonstrated experimentally by Hewlett Packard using titanium oxide with oxygen vacancies [141]. One of the main focuses of recent research in this field is to improve reliability, explore alternative methods to modulate constructed devices other than electrical modulation, and determine how the mechanism works in detail. In 2022, Mao J.Y. and co-workers presented a vertical resistive switching device comprised of Au/hBN/Au (Fig. 2a). They introduced a metal transfer technique for the top electrode, which reduces structural damage to hBN compared to the conventional metal evaporation process [109]. The device uses inert gold on both the top and bottom electrodes to ensure the intrinsic properties of hBN are the main contributor to the resistive switching behavior. The result demonstrated nonpolar resistive switching behavior for monolayers (Fig. 2b) indicating both unipolar and bipolar resistive switching behaviors in the same device. This resistive switching mechanism involves utilizing the boron vacancies in hBN. Under an applied electric field, boron (B) vacancies are redistributed, leading to the formation of conductive nanofilaments at a ‘SET’ voltage of 1.0 V, with an operating current of 1 mA. This can be seen by following the blue curve, where the device jumps from 10^− 7^ A to 10^− 3^ A indicating the transition from a HRS to a LRS. Resetting the device requires a higher current of 100 mA with a voltage of 0.6 V, which results in Joule heating that dissipates the boron vacancies removing the previously formed conductive paths. This can be seen by following the red curve, where the device returns to a HRS state as the voltage gets closer to 0.6 V. The device results in many SET/RESET cycles before degradation, indicating good stability across operation currents between 1 mA to 10 ${\upmu }$A. The device also displays fast switching time on the order of nanoseconds. In addition to this, the nonvolatile I-V characteristics of the device based on different hBN structures suggest that the thickness of the hBN layers is an important factor when considering the tunability of the resistive switching behavior. An interesting outcome of the research found that it is important to ensure there are no direct channels between source and drain through the fabrication process to ensure reliable switching and hysteresis.

A planar 2D memristor using ReS_2_/WS_2_ heterostructure was reported in 2023, showcasing unique unipolar characteristics [142]. The switching mechanism in this device is also driven by vacancy migration, particularly involving sulfur (S) vacancies in ReS_2_. These vacancies can be created with low energy due to the weak covalent bonding between rhenium (Re) and S, enabling efficient switching performance at low operational energy levels. The ‘RESET’ process occurs through Joule heating, and returns the device to a high resistance state, by redistributing the S vacancies. The authors also demonstrate how the lateral channel length of the device contributes towards the ‘SET’ voltage of the device, which allows for a device with configurable parameters. The developed device shows unipolar resistive switching, which is different from the usual bipolar switching behavior. In addition, it has a high ON/OFF ratio of up to 10^6^, and endurance and retention rates that are superior to pure ReS_2_ and WS_2_ devices. Another significant advantage of this configuration is the optical tunability of the device. In Fig. 2c without light illumination, the device operates at a normal SET voltage of 2.9 V. Under a 690 nm laser, electrons are only excited from the ReS_2_ as shown in Fig. 2d, and this produces no change in the SET voltage. Under a 532 nm excitation, the set voltage drops progressively from 2.9 V to 0.9 V by varying the optical power density from 0 to $1.1\times {10}^{-5}$ mW $\mu$m^− 2^ (Fig. 2e).

In recent years, there has been research into precise control of resistive switching devices by exploring how defects and vacancies directly affect performance. One approach presented a novel approach to understanding resistive switching in memristors by using non-destructive optical spectroscopy to track the motion of oxygen vacancies in real time [143]. The technique was used to study strontium titanate memristor films, revealing that nanoscale oxygen bubbles form on the surface of the film. These oxygen bubbles eventually lead to device breakdown on cycling. As the oxygen vacancies accumulate, they eventually develop into O_2_ gas, which perturbs the expected response.

### Polarization induced resistive switching

**Fig. 3:**
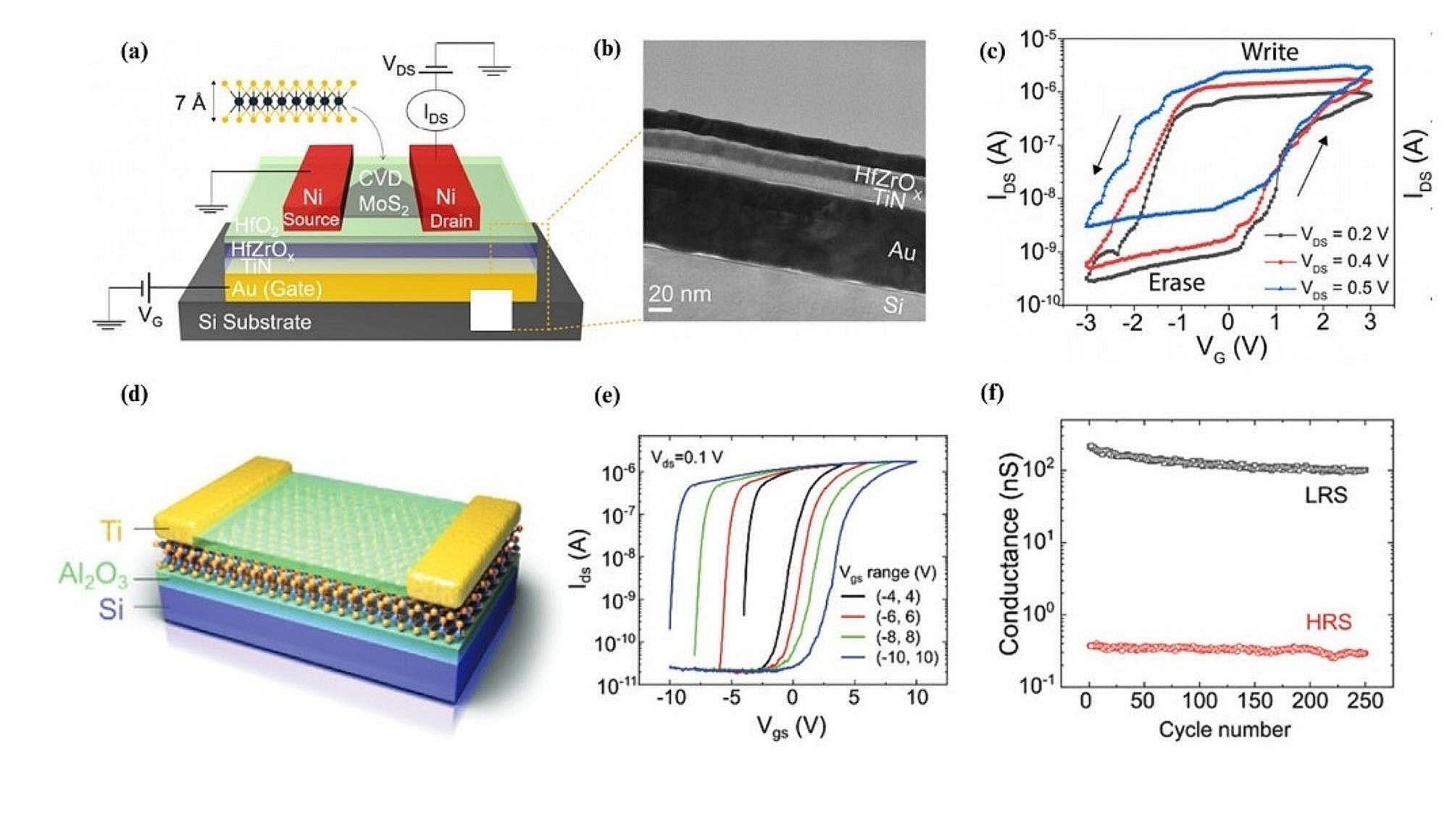
Polarization induced resistive switching as a main mechanism in 2D materials based memristors. (**a**) Schematic diagram showing the single layer CVD grown MoS_2_ used as the transistor channel, placed on top of the stack of thin film materials. (**b**) A cross sectional SEM view of a sample showing layered structure and thickness. (**c**) I-V curve of the device measured at room temperature with varying drain-to-source voltages (V_DS_). (**d**) Schematic of the device configuration surrounding the ${\upalpha }$-In_2_Se_3_. (**e**) Hysteresis curve results across a range of applied voltage values (**f**) Conductance measurement over 250 cycle numbers which demonstrates the stability of the HRS and LRS of the device. Figure 3. (**a-c**) Reproduced with permission [144]. Copyright 2020, AIP Publishing. Figure 3. (**d-f**) Reproduced with permission [145]. Copyright 2020, Wiley-VCH GmbH

Polarization induced resistive switching refers to the use of ferroelectrics, where an electric field induced change in the polarization state leads to a reversible alteration in the resistance of a material. It usually involves a ferroelectric layer of a perovskite oxide material along with a target 2DM in between two electrodes. An applied electric field can alter the direction of polarization leading to changes in the distribution and mobility of charge carriers. Advancements in nonvolatile storage include a recent study in 2020, conducted on ferroelectric field effect transistors utilizing MoS_2_ as a resistive switching channel [144]. This work presents a device utilizing MoS_2_ grown via chemical vapor deposition (CVD) to overcome the limitations of scalability and low writing voltage operation in devices that use mechanically exfoliated flakes or organic ferroelectrics. The study introduced a hybrid device as shown in Fig. 3a, consisting of a silicon substrate, a gold contact gate, TiN, HfZrO_x_ as a ferroelectric layer, and HfO_2_ as a passivation layer along with a nickel source and drain. A cross-sectional TEM image of a device is shown in Fig. 3b. The device has three different states; a LRS, an intermediate state, and a HRS. When a positive gate voltage is applied, the electric polarization of the ferroelectric HfZrO_x_ layer is directed toward the MoS_2_ channel. This results in electron accumulation in the MoS_2_ channel, leading to a high drain current, turning the device into its LRS. The LRS state is retained after the voltage is removed, as the HfZrO_x_ layer remains polarized, maintaining a locally positive electric field on the channel. Once a sufficient negative gate voltage is applied, the ferroelectric polarization changes direction. This change depletes electrons away from the MoS_2_ channel, resulting in the HRS state. A notable feature of the result is its low voltage-driven operation with a set voltage of 3 V, along with the thin HfO_2_ layer that plays a crucial role in maintaining the ferroelectricity of the device and lowering the threshold switching voltages. The result also shows multilevel conduction states through repeated fixed interval pulses, indicating the ability of the device to emulate synaptic potentiation and depression. The device demonstrated stable bipolar switching characteristics at several different drain to source voltage values as shown in Fig. 3c.

In the same year in 2020, Wang et al., reported ferroelectric switching using $\alpha$-In_2_Se_3_ sandwiched between Ti and Al_2_O_3_ as shown in Fig. 3d [145]. This material shows promise with its robust room-temperature ferroelectricity, even in monolayer form, and is recognized for exhibiting both out-of-plane and in-plane ferroelectricity. The study delves into the in-plane ferroelectric switching behavior, particularly in the absence of a gate. The resistive switching mechanism observed is attributed to the modulation of Schottky barriers by the ferroelectric polarization switching within the $\alpha$-In_2_Se_3_ channel. The device was fabricated on degenerate p-doped Si substrates, covered with a 285 nm thick layer of thermal SiO_2_ or with a 50 nm thick layer of Al_2_O_3_ deposited using atomic layer deposition (ALD). $\alpha$-In_2_Se_3_ flakes were mechanically exfoliated from a bulk single crystal onto these substrates. For the electrodes, 15 nm of titanium (Ti) was deposited followed by 65 nm gold (Au). A 15 nm Al_2_O_3_ capping layer was synthesized onto the device via ALD. The device exhibits tunable hysteresis behavior through varying the gate voltage across the device, resembling conventional ferroelectric field effect transistors. The tunable SET and RESET voltages of the device can be observed in Fig. 3e for different sets of gate voltages. In addition, the device exhibits a high ON/OFF ratio between the HRS and LRS states, exceeding 10^3^ (Fig. 3f) and possesses robust and repeatable multi-cycled resistive switching behavior which alleviates issues like interfacial charge trapping and gate leakage current which is partially accounted for by its material composition. The results present nonvolatile and volatile memory behaviors that would be useful in downstream neuromorphic computing applications such as long-term potentiation (LTP), long-term depression (LTD), and spike timing dependent plasticity (STDP). One interesting aspect of the results was the focus on both in-plane and out-of-plane resistive switching. The authors presented the in-plane resistive switching mechanism, which resulted in an unconventional doubled LRS HRS, where the resistive switching loops are found to trace each other, implying the volatility of the HRS state.

### Schottky barrier modulation

**Fig. 4:**
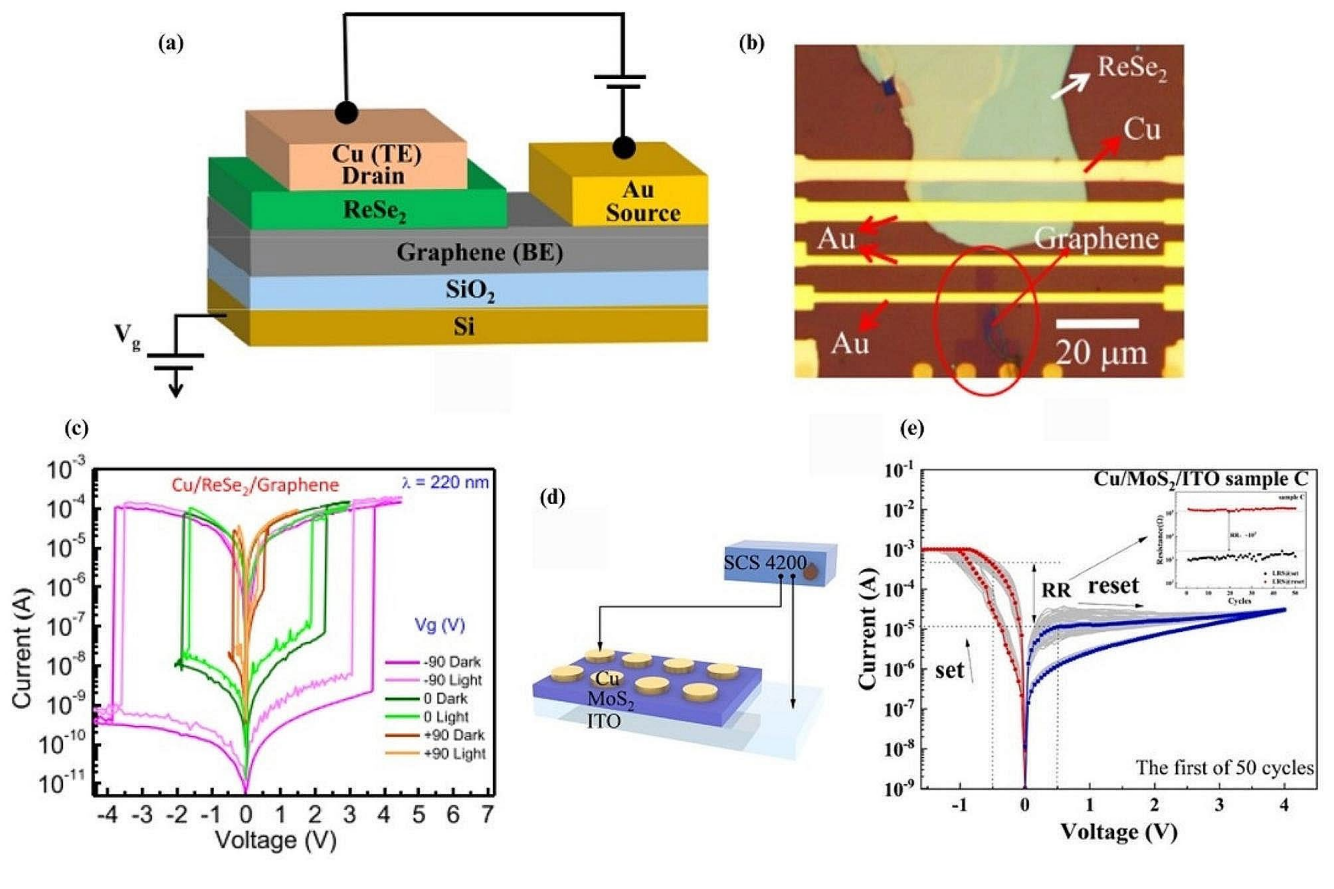
Memristive device that utilizes Schottky barrier height modulation as a resistive switching mechanism. (**a**) Schematic diagram of the ReSe_2_ memristor. (**b**) Top-view optical micrograph image of the device layer. (**c**) I-V response curve of the Cu/ReSe_2_/graphene device under different gate voltages with and without deep ultraviolet illumination. (**d**) Schematic of the Cu/MoS_2_/ITO device. (**e**) I-V curve of a several hundred nanometre thin film MoS_2_ device displaying nonvolatility. Figure 4. (**a-c**) reproduced with permission [32] Copyright 2020, Elsevier B.V. All rights reserved. Figure 4. (**d, e**) reproduced with permission [147]. Copyright 2023, Elsevier B.V. All rights reserved

One interesting demonstration in resistive switching which modulates Schottky barrier height to control the formation and dissolution of copper (Cu) ions within the ReSe_2_ was presented using a vertical copper/ReSe_2_/graphene heterostructure device as shown in Fig. 4a [32]. The top-view optical micrograph image of the device is shown in Fig. 4b. The device exhibits gate-controlled resistive switching, where the work function of graphene is electrostatically tuned, impacting the height of the Schottky barrier. This modulation leads to a variation in current levels and ‘SET’ voltages under different gate voltages. Another prominent result lies in the effect of deep ultraviolet light illumination on the device. By illuminating the device at a wavelength of 220 nm with a varying gate voltage, a clear shift in ‘SET’ and ‘RESET’ voltages was observed. This can be seen clearly in Fig. 4c, where light application along with a negative gate voltage resulted in a reduction in switching voltage from approximately 3.5 V to 3 V. Being able to vary device parameters externally using light provides opportunities for more complex operations in optoelectronic systems, due to the device having two configurable inputs (light and electricity), while maintaining low power and a high ON/OFF ratio, resulting in a robust device that has the potential for many applications. Similar switching mechanisms utilizing modulation of Schottky barriers have been reported in TMD devices in general [146], however, the use of a monolayer graphene as a bottom electrode to tune the work function through electrostatic gating could be explored in other TMD systems to discover more exciting opportunities.

In 2023, Lei et al. investigated the interplay between material thickness and volatile and non-volatile switching behavior through Schottky barrier height modulation [147]. The authors created samples of devices consisted of ITO thin films, MoS_2_, and copper thin films of different thickness as shown in Fig. 4d and measured their hysteresis cycles (Fig. 4e). They observed that the device is less likely to maintain its LRS state when the number of 2D layers are reduced. This is due to electrons escaping from the conduction band and filling trap sites in the absence of a bias voltage. This process increases the Schottky barrier height over time and causes the device to revert to its HRS. This process takes longer to occur the more layers there are of material, hence prolonging any resistive state a device may be in. However, this provides interesting insight into fabricating devices that hold their state for a fixed amount of time which can be utilized to create synapses for neuromorphic computing.

### Phase transition

Phase-change materials for memristors have been of interest in recent years due to their fast switching time and scalability. 2D materials with phase transitions hold great promise for shrinking device sizes. In recent years, there has been emerging research into phase change devices constructed from the MXene class of materials [31, 33, 35, 148]. MXenes are often incorporated with other materials in order to produce reversible phase change effects. One result in particular illustrates the use of ferroelectric MXene with reduced graphene oxide electrodes in order to show non-volatile bipolar switching [149]. The authors note the tunability of the device through heat and strain which can be changed during device preparation. The result reports an operating voltage less than 3V, with an ON/OFF ratio greater than 10^3^, a retention time of 4000 s, along with an endurance of up to 1000 cycles. Another paper proposed a switching device consisting of Ti_3_C_2_ nanosheets which displays both analog and digital resistive switching [35]. This device shows an ON/OFF ratio of 10^4^ at a switching voltage slightly less than 6 V, along with an endurance of 2000 cycles and a retention time of 10000 s.

**Fig. 5:**
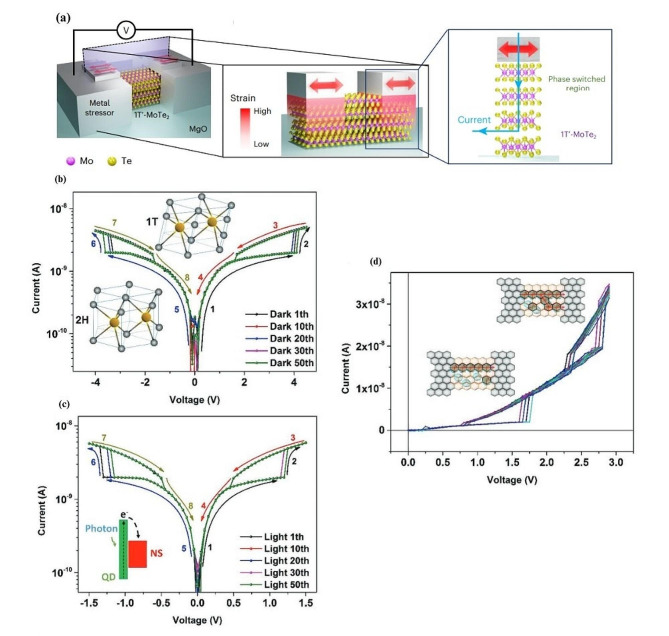
Memristive devices that use phase transition as their main mechanism. (**a**) (left) Device schematic showing stressed contact metals to MoTe_2_, (centre) cross section of the device with the strain profile, (right) the mechanism of operation for vertical transport based on phase switched MoTe_2_ beneath the contact metal. (**b**) The current voltage characteristics of the MoS_2_ quantum dot nanosheets (QDNS) structure for 50 cycles with no illumination with schematic representation of the 2H-1T phase transition from trigonal prismatic to octahedral structure. (**c**) The current voltage characteristics of the MoS_2_ QDNS structure for 50 cycles with illumination, with a schematic diagram of the process of photoexcited electron transfer. (**d**) An example of the two level resistive switching of the graphene/MoS_2_ QDNS/graphene structure. The inset schematic also represents the formation of filaments from the 1T phase. Figure 5. (**a**) Reproduced with permission [151]. Copyright 2023, The Author(s), under exclusive licence to Springer Nature Limited. Figure 5. (**b-d**) Reproduced with permission [72]. Copyright 2019, WILEY-VCH Verlag GmbH & Co. KGaA, Weinheim

A more established field of research regarding phase change materials for resistive switching are TMDs. In the context of resistive switching, molybdenum ditelluride (MoTe_2_) has strong potential for being a useful switching device, as its DFT-computed energy between its semiconducting (2H) and semi-metallic (1T′) state is only 43 meV [67–69], indicating that its phase change can be readily achieved by tensile strain, laser irradiation, and electric fields [150]. Very recently, a phase-change memristor using MoTe_2_ has shown extremely high performance surpassing that of other 2D phase-change memristors [151]. Figure 5a shows a schematic of the device, with the left image showing stressed metals in contact with the MoTe_2_, the centre image takes a cross section of the device with the strain profile across the device, showing the strain is highest closer to the contacts, and the right image illustrates the mechanism of operation based on the phase switched MoTe_2_. They implemented a strain-engineering technique in order to lower the energy difference between the semi-metallic and semiconducting states of the material, resulting in a smaller electric field required to operate the memristive switch. The paper reports switching voltages of 90 mV, ON/OFF ratios of 10^8^, switching times of 5 ns, and retention times up to 10^5^ s. One significant result that aids in the tunability the device is the ability to change the physical film stress in order to increase the ON/OFF ratio past the natural limits of the material.

In other work, phase transition in MoS_2_ is used to demonstrate a memristive device [71, 72]. This work found that when intercalated quantum dots (QDs) were illuminated, they excite electrons that polarize MoS_2_ nanosheets causing a phase transition from 2H to metallic (1T). As a result, a reversible photoinduced 2 H-1T phase transition was found in graphene/MoS_2_ quantum dot nanostructures, resulting in resistive switching of the structure upon photoexcitation of the quantum dots. The transfer of electrons from QDs to the nanosheets is observed under light illumination with low power density (0.2–0.51 mW ${\upmu }\text{m}$^−2^). This induces a phase transition from 2H to 1T which results in a blue shift along with several additional Raman scattering peaks. The switching behavior can be seen by the process labelled “1” and “2” in Fig. 5b. The phase transition from 2H to 1T is also shown to be reversible when decreasing the power density of the light to a lower value, causing redshifts and 1T MoS_2_ peaks to disappear, which is seen by the process labelled “3” and “4”. To stabilize the 1T phase after it has been achieved, a bias voltage of 4 V is applied. Experimentally, this results in an increased current with metallic conductivity indicating a stable phase transition. Lowering the bias voltage to 1.8 V turns the device off, and the electrons in the QDs are not excited enough to stabilize to the 1T phase and the 2H phase is recovered. When the structure is illuminated, resistive switching is observed at approximately 1.2 V, compared to 4 V due to the additional transfer of photoexcited electrons from the QDs to the nanosheets (Fig. 5c). Another interesting result was found by increasing the density of QDs. By increasing the amount of QDs, it increases the number of electrons supplied and hence can increase the localized region of the metal phase. In Fig. 5d, the results show a second switching level, with linear behavior for the device until 1.6 V where it exhibits nonlinear behavior, which indicates an additional local 2H-1T phase transition. The inset for the figure also shows the formation of several 1T paths for electron travel which is responsible for the multi-leveled effect.

### Charge trapping

The charge trapping mechanism involves creating a sandwiched structure with a semiconductor, an insulating layer called a tunnel oxide, a charge trapping layer, and a control gate. A voltage is applied at the control gate, which creates an electrical field that causes electrons to tunnel through the tunnel oxide into the charge trapping layer. Once the electrons have tunneled through, they get trapped in the charge trapping layer, which is usually engineered to have traps or defects where electrons can be stored for long periods. Vu et al. designed a high ON/OFF ratio memristor array to perform the resistive switching process by using the charge trapping mechanism [152]. The designed device is created using a heterostructure composed of MoS_2_/Al_2_O_3_/graphene in a vertical stack, combined with a planarized electrode structure, where the source electrode was made of chromium (Cr) and the drain electrode out of gold (Au). The device’s vertical layers were deposited using CVD, which was important to their overall functionality. The charge trapping mechanism works through electron tunnelling and storage of the charge in the graphene floating gate, where the electrons tunnel through the Al_2_O_3_ insulator. The final manufactured device produces an ON/OFF ratio of over 10^3^, with an average value of 10^4^. It also produces a reliable endurance of over 8000 cycles, and a low on and off current operating range between 10^− 6^$\text{A} {\upmu }{\text{m}}^{-1}$and 10^− 15^$\text{A} {\upmu }{\text{m}}^{-1}$.

To summarize the information regarding switching mechanisms, a comparison of recent 2D material based memristor device architectures is provided in Table 1.

**Table 1:** Memristive devices based on 2D materials

Device Structure	Switching Mechanism	Material Structure	SET Voltage (V)	ON/OFF Ratio	Retention Time (s)	Endurance (Number of Cycles)	Refs.
Vertical	Conductive Filament (ECM)	Cu/MoS_2_ double layer/Au	0.25	2.5	> 1.8.10^4^	> 20	[136]
Vertical	Conductive Filament (VCM)	LaMnO_3_/rGO nanocomposite	9.7–12.1	2–5	> 10^3^	> 100	[138]
Planar	Vacancy Migration	Au-ReS_2_/WS_2_-Au	0.4–4.5	10^6^	> 10^4^	100	[142]
Vertical	Polarization Induced	Au/TiN/HfZrO_x_/HfO_2_/MoS_2_/Ni	3	> 10^3^	-	> 10	[144]
Vertical	Schottky Barrier Modulation	Au/MX_2_/Au	< 0.6-3	100–10^7^	10^6^	150	[146]
Planar Hybrid	Phase Transition	2D Nanosheets/0D Quantum Dot MoS_2_	1.15-4	2	-	50	[72]
Planar	Charge Trapping	Graphene/Al_2_O_3_/MoS_2_/Cr/Au	8	~ 10^3^ up to 10^8^	10^3^	6×10^3^	[152]
Vertical	Conductive Filament (ECM)	ITO/CdPS_3_/Ag	0.93 (2 × 10 ms pulses)	50	> 10^3^ (pulsed input)	100	[153]
Vertical	Conductive Filament	Au/Bi_2_O_2_Se/Ag	1.8	10^3^	3000	80	[22]
Vertical	Phase Transition	rGO/FE-MXene/rGO	-3.29 to -1.65	< 10 to 10^3^	4000	10^3^	[149]

## Applications of alternative computing paradigms

### Neuromorphic computing

Conventional computers often use von Neumann architectures to process data, which involves fetching data from separate storage units and passing it to a processing unit. Approaches to mitigate this transmission issue include the development of graphical processing units (GPUs), which parallelize information to pass between storage units and their internal processing units, allowing for a significantly higher number of operations in a clock cycle. However, these devices are limited by transmission lines which continuously fetch and update data every clock cycle. In addition to this, information is often stored in binary through the conversion of an analog voltage signal to a digital representation.

Memristive devices hold significant advantages over these traditional architectures in the realm of neuromorphic computing due to their potential to emulate synapse-like behavior in artificial neural networks (ANN). When manufactured into memristive components, these devices have the ability to both store and manipulate data and weight information, making them convenient for representing synapses in ANNs.

Another common issue with software based neural networks is floating point number representation. A common phenomenon that has been reported is the vanishing gradient problem [154, 155]. This issue stems from the activation functions that certain neural networks use that push floating point representations of numbers to extremely low values [156]. After these numbers become too small, they can no longer be quantized, resulting in the value becoming zero, or requesting a larger storage unit from the memory unit, both issues being problematic. Memristive devices may have a solution to this problem through the use of analog representations of numbers.

Duan et al. utilized the analog behavior of memristors in ANNs demonstrating a three layer neural network showing an accuracy of 92% on digit recognition tasks from the popular MNIST dataset [157]. The authors utilize a two terminal MoSe_2_ device with multiple continuous resistive states allowing for good representation of weights between successive neurons. Interestingly, to show the network possesses single neuron learning capabilities, the devices are also studied as artificial nociceptors, which is a unique result not commonly reported for devices based on 2D materials.

Aside from implementations attempting to mimic ANNs, researchers have made significant progress to instead model Spiking Neural Networks (SNN) as they more closely mimic biological behaviors, leading to more effective systems for human automation. SNNs operate by changing their response based on discrete spikes rather than continuous values. These networks can be efficiently modelled by neuromorphic devices by changing the resistance of a device based on the timing of an obtained signal. As memristors are devices whose resistance depends on their past electrical history, they can efficiently mimic this behavior. In order to realise this, metrics have been developed, namely Short Term Potentiation/Depression (STP/STD), Long Term Potentiation/Depression (LTP/LTD) and Spike Timing Dependent Plasticity (STDP). These metrics represent the ability of a device to exhibit short term resistance modulation that decay quickly, long term resistance modulation that takes a longer time to decay and timing dependent changes that affect resistance depending on when a signal is received. In recent years, there has been a focus on exhibiting these synaptic effects on a variety of memristive structures. Recently, Mahata and colleagues demonstrated STP and LTP effects in their Al_2_O_3_/PdSe_2_ device [158]. The authors also demonstrated Paired Pulse Facilitation, indicating that some form of STDP was able to be achieved. Another paper by Jo and colleagues presents an artificial neural network physically constructed by creating a transformation between an ANN and SNN [159]. They construct this device by layering hBN films in between gold electrodes. They also display the STDP effects of the fabricated device which are needed in order to implement the neural networks. This implementation bridges the gap between hardware and software implementations and applications of neural networks, while preserving the beneficial properties of neuromorphic computers.

### Crossbar arrays

In recent years, there has been a significant effort in developing devices that can perform neural network operations in hardware. Specifically, the three operations of interest are addition, multiplication, and a nonlinear activation function. These operations are calculated in large matrix operations, and a proposed structure that can do these sorts of operations has been crossbar arrays.

2D crossbar arrays are nanoscale structures where two sets of parallel conductive lines intersect perpendicularly, forming a grid. At each intersection or node, a memristor is placed. By applying voltage across a set of electrodes, the resistance state of the active material at each junction can be modulated. This allows an array to store information or perform computation. Furthermore, for neuromorphic applications, memristors at each junction can emulate specific synaptic behavior. An example of this is LTP and LTD. Some devices combine both functions to create STDP capabilities, relying on timing dependence in order to weaken or strengthen interconnections.

**Fig. 6:**
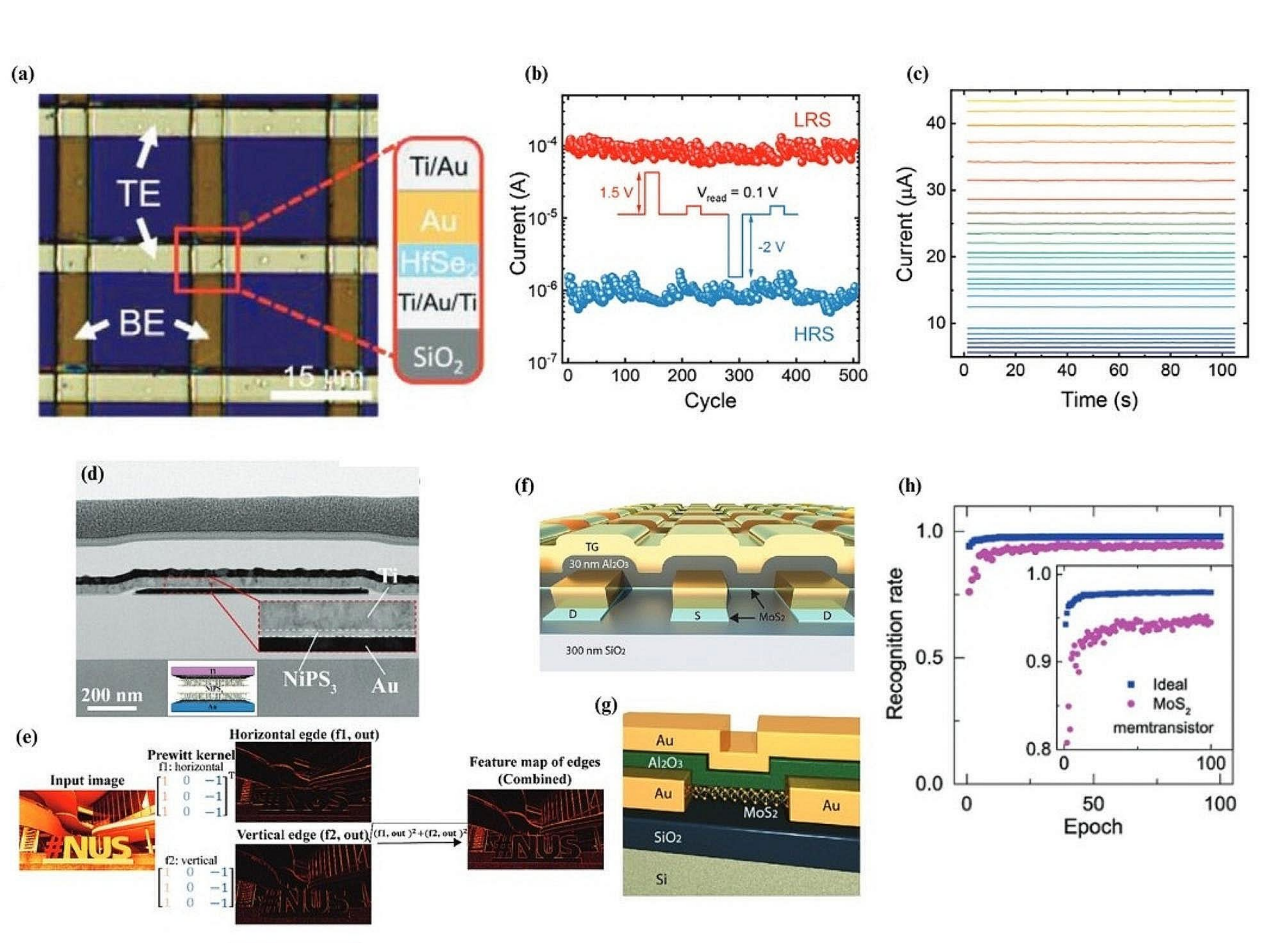
2D memristive crossbar array implementations and results. (**a**) Optical microscope image of a crossbar array including the material composition at the crosspoint region representing a single memristive unit. (**b**) Current retention of the device showing the transition from LRS to HRS over 500 cycles. (**c**) Retention of 26 different conductance states during the analog transition from HRS to LRS. (**d**) Schematic and TEM image of a fabricated Ti/NiPS_3_/Au memristor. (**e**) Horizontal and vertical edge detection kernels programmed into the circuits to perform an edge detection function. (**f**) Schematic illustration of a dual gated MoS_2_ memristor crossbar array. (**g**) Schematic of the dual gated MoS_2_ memtransistor structure. The Si acts as a global bottom gate, and patterned Au acts as a local top gate. (**h**) Recognition rate of a simulated ANN using experimental data from the memtransistor LTP and LTD values. Figure 6. (**a-c**) Reproduced with permission [19] Copyright 2021, Wiley-VCH GmbH. Figure 6. (**d, e**) Reproduced with permission [160]. Copyright 2023, Wiley‐VCH GmbH. Figure 6. (**f-h**) Reproduced with permission [161]. Copyright 2020, Wiley‐VCH GmbH

One piece of research that focused on developing a crossbar array using 2D hafnium diselenide (HfSe_2_) and developing fabrication techniques to improve performance of switching voltages also shows the performance of such devices on neural network optimization problems [19]. Figure 6a displays an optical microscope image of one unit cell of the crossbar array, with the layers vertically stacked. The transfer method developed uses a metal assisted van der Waals process to avoid direct contact with HfSe_2_ and issues with water contact. The qualitative results of the process show a negligible effect on film roughness, indicating that the process is suitable for developing large scale crossbar arrays. The individual memristors were tested to ensure resistive switching behavior, where the endurance feature is especially important in practical neural network implementations. It was found that the device had an excellent SET voltage around 0.6 V along with small power usage of 0.82 pJ. It was also found that a retention time of 10^4^ seconds was obtained with minimal current decay over many cycles (Fig. 6b). The authors also use conductive atomic force microscopy to determine that the cause of the resistive switching can be attributed to conductive filament formation. The results provide several demonstrations of synaptic functions important to biological neural networks such as LTP and LTD. The authors also determined that the conductance of intermediary resistive states between LRS and HRS were stable which may be useful for downstream applications in multilevel information representation (Fig. 6c). To validate the practicality of their approach, the team also simulated the hardware in software and trained the system on the MNIST dataset. In this case, they used a system that took an image and translated it to a 400-input neural network, with 100 hidden neurons, and 10 output neurons. The result from their simulation predicted that their system would achieve a recognition accuracy of 93.34%.

Another piece of alternative research created a crossbar array based on 2D layered nickel phosphorous trisulfide (NiPS_3_) with titanium and gold electrodes notably demonstrating the material’s ability to achieve multilevel resistance states [160]. A transmission electron microscope (TEM) image of the device along with its schematic structure is shown in Fig. 6d. The 2D material chosen here inherently had a desirable operating voltage less than 1 V, a fast switching speed along with a modest ON/OFF ratio greater than 10^2^. It is reported that the switching behavior of this device is related to conductive filaments made of Ti, along with the drift of phosphorous-sulfur ions, leading to vacancies allowing for easier formation of conductive filaments. The resulting heterostructure was 6 nm thick, and the results verify that the thinner structure was significant in obtaining high performance and uniformity. As with other research into crossbar arrays, the obtained results were simulated, and an ANN image recognition system was constructed to verify the obtained result. Interestingly from this research, the crossbar arrays were used to recognize an image in a form of feed-forward calculation of the crossbar array. Figure 6e shows an input image that is converted into its horizontal and vertical edge constituents using the crossbar array. This proved the system could perform multiply and addition functions effectively, a strong step towards showing that these devices can work in practice given the correct weights.

Other research has focused on designing memristive crossbar systems that introduce a second gate terminal, in order to tune their device weights in real time [161]. The research utilized monolayer MoS_2_ with two gate terminals in order to fine tune the state of the memristive device (Fig. 6f, g). This allows the device to tune its resistance dynamically based on a learning rate [162]. Through this system, they have also improved the performance of each individual memristor by reducing an aspect referred to as sneak current, where current flows through adjacent memristor nodes, affecting their resistance readout [163]. The authors also present a hardware demonstration of the implementation of the ANN, which is important to lead to future work in implementing the entire system in hardware. The paper demonstrates the capabilities of the array by simulating an artificial neural network and demonstrating the potentiation and depression of the system. Figure 6h shows a comparison between a software neural network and experimental data from the crossbar array. The performance is comparable, although it never achieves perfect accuracy.

An area of significant importance in the construction of 2D crossbar arrays is intra device variability during construction. Mechanical exfoliation is commonly performed for the active area of memristor devices, which leads to variability in performance and a lack of scalability. Recently, attempts have been made to construct hBN devices with low variability through chemical vapour deposition, and the results have shown a yield exceeding 98%, cycle-to-cycle variability of 1.53%, and device-to-device variability of 5.74% [16]. In fact, hBN has been shown to have one of the lowest device-to-device variance and highest yield out of all 2D material made memristors due to the dominance of the most conductive defects present in its structure, resulting in a lower sensitivity during device fabrication and resistive switching processes [164]. One result that presents an improvement utilizes HfSe_2_ on a crossbar array grown by molecular beam epitaxy, along with a metal assisted van der Waals transfer technique [19]. The reported result exhibits a low switching voltage while being able to replicate synaptic properties present in SNNs. The reported result also mentions cycle-to-cycle variability of 2.42%, while also reporting accuracy on MNIST datasets as 93.34%. Another recent result presented the synthesis of 2H-MoTe_2_ with vertically aligned grain boundaries in order to create a device with more uniformity across its fabrication [165]. The MoTe_2_ was fabricated using chemical vapour deposition, and the results obtained show cycle-to-cycle variation of 8.3%, a yield of 83.7% with device variability between 8.3% and 14.2%. Another exciting area of research is the development of new techniques for fabricating Janus 2D materials [166]. The new method developed called chemical breakdown, can obtain large area uniform Janus graphene oxide films, which can open new avenues for the development of reliable crossbar arrays using this material. A demonstration of the material in a memristive device is presented as well which obtains a very low operation voltage of 0.3 V with an extremely high endurance for 2D materials, exceeding 12000 cycles.

Crossbar arrays have also shown improvements in recent years with respect to large scale integration and power efficiency. Recently, monolithic three dimensional hetero-integration technology was proposed for stacking functional layers of a crossbar array vertically [164]. A total of six layers of transistor and memristor arrays were vertically integrated into into a 3D system to perform tasks related to artificial intelligence. The result shows an improvement in processing time, voltage drops, latency, and footprint due to the densely packed layers, with a further reduction in these metrics as the number of stacked layers was increased progressively. Another approach to improving integration in large arrays utilizes calibration in order to mitigate hardware imperfections causing errors [165]. The approach aims to find the difference between the ideal behavior of the device and the actual behavior device by performing current and voltage sweeps over the operating region of the device and using the result as a correction metric. Another area of interest for large scale integration is the creation of reconfigurable devices. Luo et al. proposed a logic circuit composed of one transistor and one memristor [166]. By using this scheme, logic operations can be performed on both rows and columns. The device both stores the computed information and performs the computation on each sub-array, avoiding problems such as sneak current. Finally, one of the key reasons to use memristors, and more practically crossbar arrays is for their energy efficiency. In 2024, Weng et al. proposed a 2D layered nickel phosphorus trisulfide memristive advice in a crossbar array configuration [167]. Their device obtains impressive metrics as a singular unit, having an operating voltage less than 1 V, switching speed less than 20 ns, and an ON/OFF ratio of 10^2^, with the energy efficiency for a multiply accumulate operation of 56 TOPS/W, which is significantly better than commercially available devices which obtain efficiencies between 5 and 20 TOPS/W [168].

## Conclusion and perspective

Devices utilizing memristive technology have been a popular area of research for many years now. The technology has matured as a building block to bypass limitations present in our current computing architectures for future applications such as neuromorphic computing. Despite this, there is significant progress that needs to be made for further research and commercialization of memristive devices.

There are several mechanisms to explain how resistive switching works in device architectures, but the transport mechanisms are not well understood. This results in unclear definitions and overlaps in explanations for resistive switching mechanisms. The exploration of materials has also been limited to certain classes of materials, and there needs to be more research into exploring new types of materials for switching behaviors.

Investigating the mechanisms of switching behaviors is challenging because switching occurs on the nanosecond level and involves rearrangements at an atomic level with stochastic processes. The descriptions of current processes can be ambiguous and require more robust mathematical models to design better devices in the future. In addition to this, material decisions are usually based on previously successful material classes while not considering the resistive switching mechanisms themselves, and instead only considering output metrics such as switching times and ON/OFF ratios. In addition to this, switching mechanisms in 2D materials are even less understood, and explanations for why certain mechanisms operate should be explored further. Hypotheses can be made to ascertain why switching times are better in many 2D materials when compared to their bulk counterparts. For example, when considering conductive filament construction, formation of atoms occurs using a random walk [169, 170]. Random walks constrained in one dimension are proven to not diverge [171], and as a result can form paths quicker across lateral devices. This could be an explanation for the benefits of using 2D materials, but further investigation would be required.

Another area of undeveloped research is the area of using optical effects in memristive devices. There has been progress towards developing optoelectronic devices where optical modulation can be used to change resistive states and act as another degree of freedom. However, to turn these into functioning devices, an electrical component is still needed. In contrast, all optical switching devices have been limited to bulk materials [172, 173], with operating conditions being limited in such cases. There is also potential to use areas of study such as valleytronics which use similar materials to existing 2D material switching devices, such as graphene and TMDs [174–177] to add degrees of freedom and consequently create memristive devices with a larger number of states, improving the resolution of neuromorphic computing.

Despite the advancement in memristive devices in terms of their construction, material composition, and synaptic functionality, their demonstration is often as a single isolated device. In neuromorphic applications, memristors are often constructed into crossbar arrays. There has been research into proving the functionality of these devices in this configuration where they show promise, being able to maintain their memristive behavior, but research demonstrates limitations in aspects of on-chip programmability, nonlinear activation functions built into the hardware, and demonstrations of computations being limited to simplistic image recognition tasks.

For example, Li et al. built a crossbar array that demonstrates memristive behavior, and the device can perform add and multiply operations, but there is an absence of how the device will implement nonlinear activation functions [19]. In this regard, more research is needed to develop nonlinear activation functions directly into hardware. Creating these functions on a separate system would defeat the purpose of a hardware-based neural network, as at every step data would need to be transferred away from the crossbar array. In this respect, a promising area of research in the future would be the application of nonlinear functions directly in hardware for these structures [178]. However, the functions being researched are still not sophisticated enough to be applied to large-scale systems consistently.

While crossbar arrays provide a convenient method to create interconnected memristive devices, they still suffer from issues such as scalability in fabrication, and integration with existing technologies such as CMOS. 2D material based memristive devices often exhibit significantly lower endurance in SET and RESET cycles, limiting the long term usage and integration of these devices into modern accessible hardware. However, this poses an issue in utilizing these devices for applications where resistance values need to be changed a large amount of times, such as training a neural network. Recently, nanosheets of solution processed MoS_2_ memristors have been shown to possess low variability while retaining the high memory retention times of other 2D material memristors [179]. In addition to this, there are emerging large scale manufacturing methods for devices made out of Janus 2D materials for example, that possess extremely high endurance compared to existing devices [180]. Despite these isolated research attempts, wider research into material processing techniques such as these need to be explored in order to make progress into developing devices with a higher endurance. Another common issue presented in 2D material memristors is variation across different devices. In a crossbar array where there are a large amount of fabricated devices, this can affect the stability and performance. As mentioned in Sect. 4.2, in recent years there has been significant effort to alleviate these issues by replacing inaccurate manufacturing techniques such as mechanical exfoliation with chemical vapour deposition allowing for a more uniform distribution of layer thickness [16, 181]. In addition to this, there has been further developments into reducing misalignments between grain boundaries and ensuring high crystalline quality of synthesized 2D materials [21]. One promising area of research that has shown excellent conformality is the use of atomic layer deposition (ALD) [182]. Recently, there has been some research involving the deposition of MoS_2_ using a commercially scalable ALD process [183]. Unfortunately, research in using ALD for the growth of 2D material crossbar arrays is limited, and only specific to certain materials. In order to integrate these devices into existing CMOS technologies, some work has created supplementary circuitry in order to allow devices to interact with each other [18], while other work has created new manufacturing techniques such as solution processed MoS_2_ to reduce device variability and pave the way for future CMOS integration [179, 184]. However, future integration of these devices should be monolithic, or manufactured on the same space as CMOS circuitry in order to maximize their potential. Early implementations of this idea has shown significant improvement due to the precise control CMOS circuitry can exhibit over 2D memristive devices. One paper has shown an endurance of 5 million cycles using a hybrid 2D-CMOS chip with hBN as the active material [185], far exceeding any previously reported result from standalone devices or externally integrated devices. Other device integration attempts have looked at building these devices upwards in layers, and creating interconnections through vias. While the results are limited in this case, it has been shown to reduce power consumption by 50% and the design area by 35%.

Heterostructures made from several layers of 2D materials are also an avenue to address issues like reliability across large scale integration while also integrating unique functionalities into the same device. For example, Chen et al. developed a nonlinear memory selector utilizing a heterostructure composed of MoS_2_/WSe_2_/MoS_2_, which is a property that is needed in order to fully realise neural networks while maintaining a high current density [186]. Other research shows a highly reliable heterostructure device constructed with MoS_2_/Nb_2_O_5_ in which the Nb_2_O_5_ interlayer thickness can be used as a parameter to tune the switching characteristics. The authors state the more reliable behavior is due to the increased Schottky barrier height at the 2D channel-electrode junction, which results in more effective contact barrier modulation and hence more reliable resistive switching [187]. More recently, research by Mahata et al. has also displayed the reliability of heterostructures using conductive filament, constructing a device using PdSe_2_ [158]. Their device provided reliable multilevel conductance along with improved endurance and several synaptic learning effects. Despite these advancements, one of the main issues with heterostructures is the fact that their behavior is specific to their monolayer composition, and different structures will produce different results. As a result, the accuracy and performance of a heterostructure is directly related to the precision of the manufacturing process, and hence it is possible that more unique and effective heterostructure configurations will be seen as more precise manufacturing techniques like ALD are adopted.

Evaluation of crossbar arrays is often limited to how much of a contrast they can make between their on and off states, and how reliable they are through cycle retention. These parameters can be translated into weight update functions and data representation states such as ON/OFF for a neuron, however they do not provide a clear picture of the entire functionality. For a manufactured device to be useful, it needs to be able to update in real time rather than train the network offline and update the weights progressively. Hence, an implementation of backpropagation in hardware is important for future research into neuromorphic hardware to make progress towards a pure hardware neuromorphic system.

Regarding image recognition tasks, several crossbar arrays demonstrated their effectiveness using convolutional neural networks (CNN) [188]. However, when considering the difficulty of solving a CNN problem such as the MNIST dataset [189], it should be noted that it can be efficiently categorized with high accuracy and low computational effort currently by software neural networks. As a result, it may be more beneficial to try and use hardware based neural networks on problems that software neural networks find challenging. Examples of this would be image generation tasks, which currently take large amounts of computational effort on software based neural networks [190]. Issues such as a vanishing gradient [191] and quantization [192] in software based neural networks are fundamental limitations which lead to model failures and increased compute times. In comparison, using continuous representations of numbers would allow a neural network to learn without digital limitations and produce more efficient and accurate results. Another benefit earlier discussed would be the reduced energy consumption which would be significant in the case of image generation, as these algorithms need to run the same costly computation over many iterations [193]. Evaluating the efficiency of upcoming technology using more complex methods such as image generation is needed in order to use this technology in real world applications.

## Data Availability

Not applicable.
